# Characterization of retinal microvasculature and structure in atrial fibrillation

**DOI:** 10.3389/fcvm.2023.1229881

**Published:** 2023-12-13

**Authors:** Junfeng Liu, Wendan Tao, Dayan Li, William Robert Kwapong, Le Cao, Xiaoling Zhang, Chen Ye, Shi Chen, Ming Liu

**Affiliations:** ^1^ Department of Neurology, West China Hospital, Sichuan University, Chengdu, China; ^2^ Cardiac Ultrasound Office, Department of Cardiology, West China Hospital, Sichuan University, Chengdu, China; ^3^ Department of Cardiology, West China Hospital, Sichuan University, Chengdu, China

**Keywords:** atrial fibrillation, optical coherence tomography, optical coherence tomography angiography, retinal structure, retinal microvasculature

## Abstract

**Background and objective:**

Quantitative changes in retinal microvasculature are associated with subclinical cardiac alterations and clinical cardiovascular diseases (i.e., heart failure and coronary artery disease). Nonetheless, very little is known about the retinal vascular and structural changes in patients with atrial fibrillation (AF). Our study aims to characterize the microvasculature and structure of the retina in AF patients and explore their differences in different types of AF (paroxysmal and sustained AF).

**Methods:**

This cross-sectional study was conducted at the Departments of Neurology and Cardiology in West China Hospital, Chengdu, China. Individuals aged 40 years or older with a diagnosis of AF were eligible for inclusion and underwent an evaluation and diagnosis confirmation before enrollment. Control individuals aged 40 years or older and without a history of AF, ocular abnormalities/disease, or any significant systemic illness were recruited. The retinal vascular and structural parameters were assessed using swept-source optical coherence tomography (SS-OCT)/SS-OCT angiography. Echocardiographic data of left atrium (LA) diameter were collected in patients with AF at the time of inclusion.

**Results:**

A total of 242 eyes of 125 participants [71 men (56.8%); mean (SD) age, 61.98 (8.73) years] with AF and 219 eyes of 111 control participants [53 men (47.7%); mean (SD) age, 62.31 (6.47) years] were analyzed. In our AF cohort, 71 patients with paroxysmal AF and 54 patients with sustained AF (i.e., persistent/permanent AF) were included. Decreased retinal microvascular perfusion (β coefficient = −0.08; 95% CI, −0.14 to −0.03) and densities (β coefficient = −1.86; 95% CI, −3.11 to −0.60) in superficial vascular plexus (SVC) were found in the eyes of the participants with AF. In regard to retinal structures, thinner ganglion cell–inner plexiform layer (GCIPL; β coefficient = −2.34; 95% CI, −4.32 to −0.36) and retinal nerve fiber layer (RNFL) thicknesses (β coefficient = −0.63; 95% CI, −2.09 to −0.18) were observed in the eyes of the participants with AF. The retinal parameters did not significantly differ between paroxysmal and sustained AF (all *P* > 0.05). However, significant interactions were observed between LA diameter and AF subtypes with the perfusion and densities in SVC (*P* < 0.05).

**Conclusion:**

This study found that individuals with AF had decreased retinal vascular densities and perfusion in SVC, as well as thinner GCIPL and RNFL thickness compared with age- and sex-matched control participants. The differences of the retinal microvasculature in SVC between paroxysmal and sustained AF depend on the LA diameter. Given our findings, further longitudinal studies with our participants are of interest to investigate the natural history of retinal microvascular and structural changes in individuals across the clinical process of AF and AF subtypes.

## Introduction

Atrial fibrillation (AF), which is one of the most prevalent cardiac arrhythmias in clinical practice, is associated with heart failure and ischemic stroke (1), placing important economic burden along with significant morbidity and mortality (2). Accompanying the aging of populations worldwide, the prevalence of AF is rising, affecting an estimated 6–12 million people by 2050 (3). In AF patients, cardiac output decreases by 20%–30% (4), causing reduction of blood supply to the organs and microvascular abnormalities (5), whereas the characteristics of the microvasculature in patients with AF are underexplored.

The eye offers a unique opportunity to non-invasively access the microcirculation (6). A recent cumulative evidence showed that the retinal microvascular bed has also been described as a window to the heart (7–9). In addition, recent studies showed that quantitative changes in retinal microvasculature (narrower arteriolar caliber and/or wider venules) were associated with subclinical cardiac alterations (i.e., enlarged left atrial size and left ventricular dysfunction) and clinical cardiovascular diseases (i.e., heart failure and coronary artery disease) (10, 11). All the above evidence may imply a similar hemodynamic and microvascular changes between the heart and the eye.

So far, few studies have investigated the relationship between retinal vascular changes and AF (12, 13), especially their associations with different types of AF (e.g., paroxysmal and persistent/permanent AF). In addition, previous studies reported that patients with persistent/permanent AF had larger left atrium (LA) volume compared with paroxysmal AF (14–16). Given the influence of different AF subtypes on cardiac structures (i.e., LA enlargement) and their common involvement in hemodynamics, the interaction of these two factors is of interest to better understand how they jointly give rise to microvascular vascular functions. Therefore, we aim to investigate the characteristics of the retinal microvasculature and structure in AF patients and estimate their determinants in different types of AF [i.e., paroxysmal and sustained AF (persistent/permanent AF)]. Then, we investigate the interactions between LA diameter and AF subtypes on retinal parameters. We first hypothesize that AF patients, especially sustained AF, are likely to have decreased retinal vascular perfusion/densities and thinner retinal structures. Second, patients with sustained AF are more likely to have retinal vascular and structural impairment, and the differences of retinal parameters between paroxysmal and sustained AF depend on the LA diameter.

## Methods

This cross-sectional study was approved by the Biomedical Research Ethics Committee of West China Hospital, Sichuan University [2020 (104)] and followed the tenets of the Declaration of Helsinki. Written informed consent was obtained from all eligible individuals before enrollment.

## Study participants

Individuals with a diagnosis of AF between March 2021 and May 2022 were recruited from the Department of Cardiology, West China Hospital, Sichuan University, Chengdu, China. All individuals with AF were evaluated by an experienced cardiologist prior to enrollment, and the clinical diagnosis of AF was based on the results of a rhythm record with 72 h continuous recording. According to a previous study (17), AF was then classified into paroxysmal and sustained AF which was defined as the combination of persistent AF and permanent AF. We included patients with persistent and permanent AF in a single group since these subtypes are often not distinguished during consultation.

The inclusion criteria of AF patients were as follows: (1) aged 40 years or more and (2) no previously diagnosed stroke and thromboembolic event. Subjects who refused or could not cooperate with retinal examination or have a history of ocular surgery or ocular diseases (e.g., macular degeneration, severe cataract, glaucoma, and macular) were excluded.

**Figure 1:**
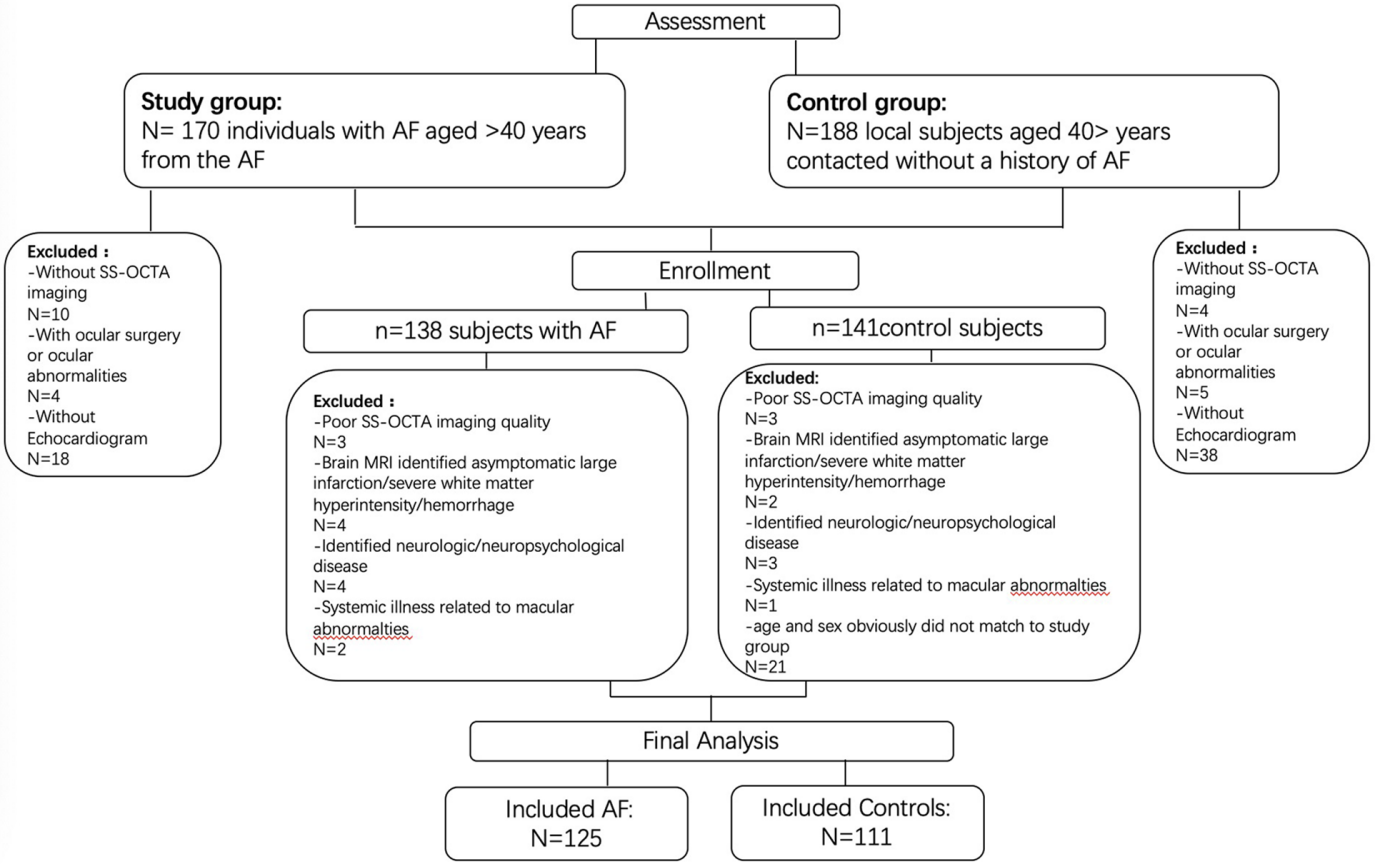
Flow diagram of the participants.

Participants were recruited from West China Hospital, Sichuan University, Chengdu, China. Control individuals were volunteers, 40 years or older, and without a history of AF, ocular abnormalities/disease (preexisting glaucoma, cataract, age-related macular degeneration, optic neuritis, high myopia, etc.) or any systemic illness that may potentially impact the structure and microvasculature of the retina such as hyperthyroidism eye disease, diabetic retinopathy, rheumatic disease, hereditary disease, or neuromyelitis optica spectrum disorders. As part of a healthy aging study, control participants underwent neurologic and neuropsychological examination with normative standards (18). Considering the influence of age and sex on the changes of retinal vasculature or cardiac structure (19–22), we excluded participants in the control group with age and sex that obviously did not match the AF group. A flow diagram of the participants is presented in Figure 1.

All participants received a clinical face-to-face interview, and a standardized questionnaire was used to collect demographic data (age and sex) and vascular risk factors (hypertension, diabetes mellitus, hyperlipidemia, alcohol consumption, and current smoking) of the participants.

## OCT/OCTA image acquisition

An experienced well-trained ophthalmologist performed all retinal imaging using the swept-source optical coherence tomography (SS-OCT) and OCT angiography (OCTA) (VG200S; SVision Imaging, Henan, China; version 2.1.016). The details of the OCT/OCTA tool were well described in our previous report (23). The retinal nerve fiber layer (RNFL), ganglion cell–inner plexiform layer (GCIPL), and inner nuclear layer (INL) were imaged in a 3 mm × 3 mm area around the fovea at the macula. Segmentation and measurement (in µm) of the retinal thicknesses were automatically performed by the OCT tool. For our study, the mean retinal thicknesses were used for all participants.

OCTA was used in imaging the retinal microvasculature of all participants in a 3 mm × 3 mm area around the fovea. En face angiograms of the superficial vascular plexus (SVC) and deep vascular plexus (DVC) were automatically segmented and measured to evaluate the retinal microvascular densities and perfusion. Segmentation of the SVC and DVC was defined as the inner two-thirds and outer one-third interface of the ganglion cell layer and inner plexiform layer (GCL + IPL). OCT/OCTA data displayed in our study followed the OSCAR-IB quality criteria (24) and APOSTEL recommendation (25). Mean density (%) and perfusion (mm^2^) were used to measure the retinal microvasculature.

The OCT/OCTA is equipped with a 3D projection artifact removal (PAR) software (PAR-OCTA, SVision, Inc.) to remove images with artifacts. In addition, images with a signal quality score of less than 7 were excluded from our study.

### Echocardiographic data collection

Echocardiographic data were collected in patients with AF at the time of the inclusion. The diameter of LA was measured using M-mode or two-dimensional echocardiography, from the posterior aortic wall to the posterior left atrial wall, in the parasternal long-axis view at the end-ventricular systole (26).

### Statistics analysis

The Shapiro–Wilk test was used to examine the normality of our data. Continuous variables are described by mean ± standard deviation (SD), while categorical variables are expressed as frequencies and percentages. Demographic variables of the participants were compared across groups using the *χ*^2^ test for categorical variables and ANOVA for continuous variables.

Linear regression with generalized estimating equation (GEE) was constructed to investigate the retinal structural and microvascular differences between controls and AF adjusting for age, gender, hypertension, diabetes mellitus, dyslipidemia, alcohol, smoking, and inter-eye dependencies. Similar analyses were performed between different AF subtypes (i.e., paroxysmal AF vs. controls; sustained AF vs. controls and paroxysmal AF vs. sustained AF).

Linear regression was further performed to test the interaction between LA diameter and AF subtypes on retinal parameters by including the cross-product term of “individual LA diameter × AF subtypes” with the main effect terms of each variable in the models. These models were also adjusted for covariates as aforementioned. All statistical analyses were conducted using the statistical software R version 3.4.1 (http://www.R-project.org). The difference was considered statistically significant if the *P*-value was <0.05.

## Results

### Characteristics of the study participants

Characteristics of the study participants were presented in Table 1 according to their diagnostic status. A total of 125 AF patients [71 men (56.8%); mean (SD) age, 61.98 (8.73) years] and 111 age- and sex-matched control individuals [53 men (47.7%); mean (SD) age, 62.31 (6.47) years] were finally included in the study. A total of 242 eyes of 125 participants with AF and 219 eyes of 111 control participants were analyzed. Of our 125 AF patients included, 71 patients with paroxysmal AF and 54 patients with sustained AF were identified.

**Table 1:** Characteristics of study participants.

	Controls (*n* = 111)	AF (*n* = 125)	*P*
Age, mean (SD)	62.31 (6.47)	61.98 (8.73)	0.74
Male, *n* (%)	53 (47.7)	71 (56.8)	0.19
Hyperlipidemia, *n* (%)	24 (21.6)	20 (16.0)	0.32
Hypertension, *n* (%)	25 (22.5)	47 (37.6)	0.02
Diabetes, *n* (%)	4 (3.6)	20 (16.0)	0.002
Drinking, *n* (%)	32 (28.8)	39 (31.2)	0.78
Smoking, *n* (%)	21 (18.9)	35 (28.0)	0.13

### Association between retinal parameters and AF

**Figure 2:**
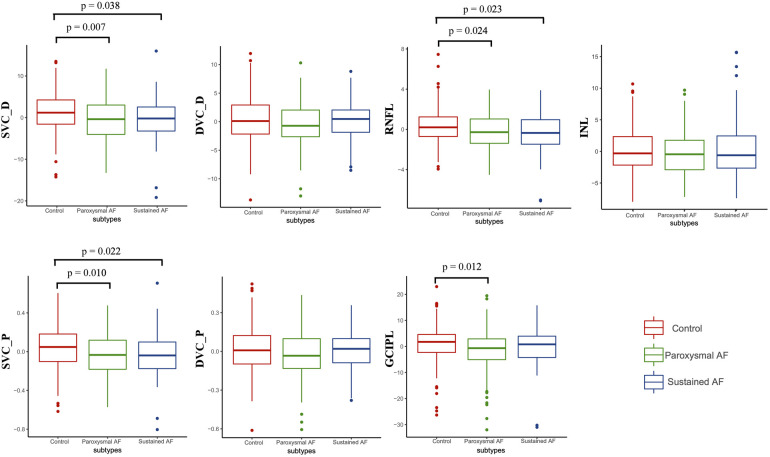
The differences of retinal parameters between controls, paroxysmal, and sustained AF. *P* was adjusted for age, sex, hypertension, diabetes mellitus, dyslipidemia, alcohol, smoking, and inter-eye dependencies. DVC_P, deep vascular plexus perfusion; SVC_P, superficial vascular plexus perfusion; DVC_D, deep vascular plexus density; SVC_D, superficial vascular plexus density.

**Figure 3:**
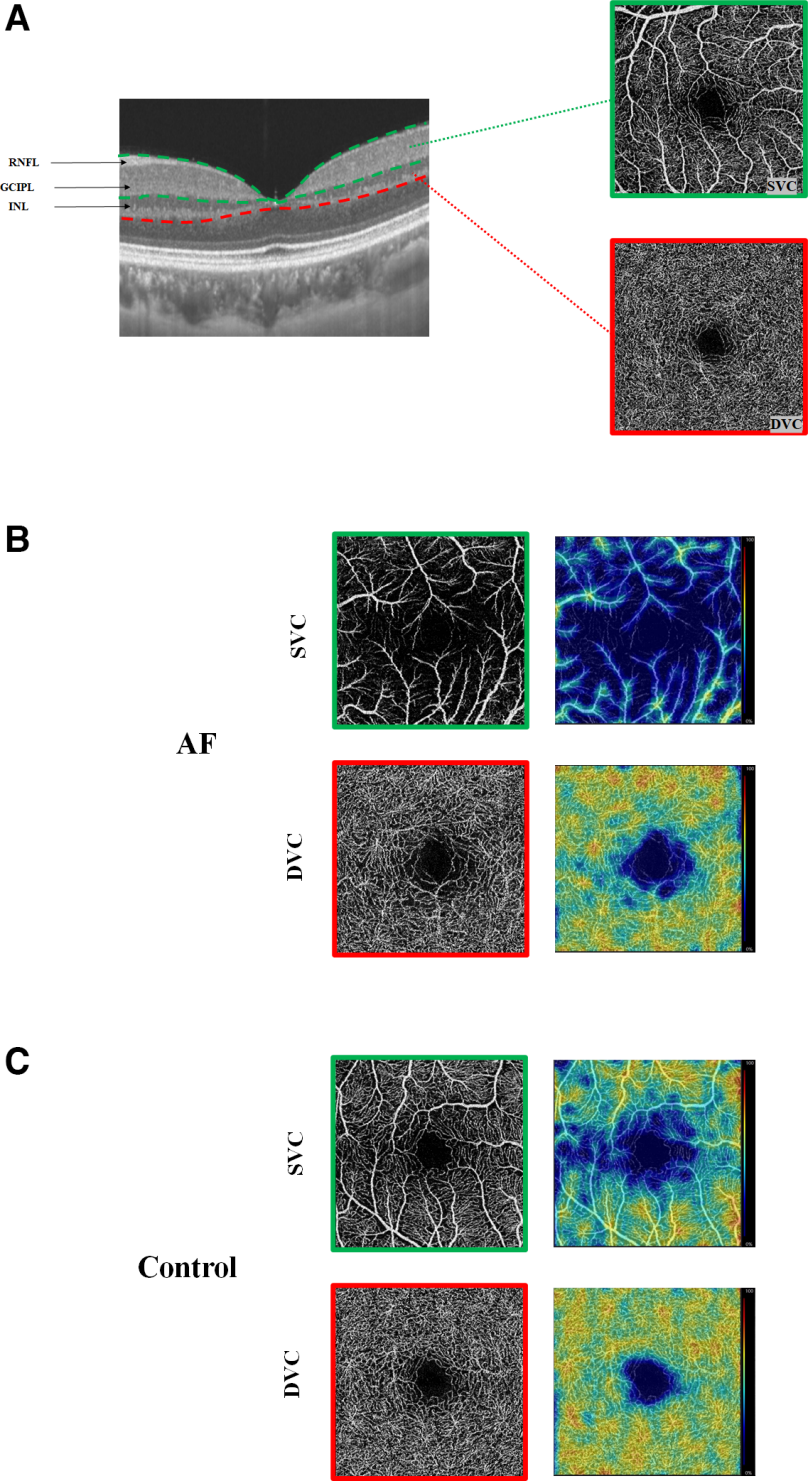
Illustrative image of the retinal structure and microvasculature. (**A**) Segmentation of the retinal structure and retinal microvasculature. (**B**) Angiograms of an AF patient (left) and color maps of the retinal densities (right). (**C**) Angiogram of a control and color maps of the retinal densities (right). With the color maps, higher retinal densities (%) and perfusions (mm^2^) are bright colors, while lower densities (%) and perfusions (mm^2^) are dull colors.

Results of a GEE analysis of the association between retinal parameters and AF are presented in Table 2. After adjusting for age, sex, and vascular risk factors, individuals with AF had decreased retinal microvascular perfusion (β coefficient = −0.08; 95% CI, −0.14 to −0.03) and densities (β coefficient = −1.86; 95% CI, −3.11 to −0.60) in SVC. The two groups did not differ with respect to the retinal microvasculature in DVC (perfusion: β coefficient = −0.03; 95% CI, −0.07 to 0.01; densities: β coefficient = −0.46; 95% CI, −1.37 to 0.45). In regard to the retinal structures, individuals with AF had both thinner GCIPL (β coefficient = −2.34; 95% CI, −4.32 to −0.36) and RNFL thickness (β coefficient = −0.63; 95% CI, −2.09 to −0.18) compared with control individuals. When we separately compared paroxysmal and sustained AF to controls, similar results were found (Table 3 and Figure 2), except that no significant differences were found between sustained AF and controls in terms of the GCIPL thickness (Table 3; β coefficient = −1.49; 95% CI, −3.86 to −0.87; Figure 2). Furthermore, after adjustment, we did not find any significant difference in INL thickness when compared with the groups (Tables 2, 3). Meanwhile, the retinal parameters did not significantly differ between paroxysmal and sustained AF (all *P* > 0.05; Table 3 and Figure 2). Illustrative image of the retinal structure and microvasculature was depicted in Figure 3.

**Table 2:** Generalized estimating equation analysis of the association of retinal parameters with AF.

Variables	Control participants (*n* = 111)	Participants with AF (*n* = 125)	β coefficient (95% CI)	*P*	β coefficient (95% CI)^a^	*P* ^a^
	Mean (SD)	Mean (SD)				
Retinal microvasculature						
DVC_P	2.32 (0.18)	2.28 (0.17)	−0.03 (−0.07 to 0.007)	0.10	−0.03 (−0.07 to 0.01)	0.14
SVC_P	1.95 (0.22)	1.87 (0.23)	−0.07 (−0.13 to −0.20)	0.007	−0.08 (−0.14 to −0.03)	0.004
DVC_D	50.65 (4.07)	50.08 (3.76)	−0.56 (−1.46 to 0.33)	0.22	−0.46 (−1.37 to 0.45)	0.32
SVC_D	40.84 (4.91)	39.17 (5.24)	−1.68 (−2.88 to −0.47)	0.006	−1.86 (−3.11 to −0.60)	0.004
Retinal structure						
GCIPL thickness, µm	77.36 (6.97)	75.29 (8.13)	−2.07 (−3.97 to −0.18)	0.03	−2.34 (−4.32 to −0.36)	0.02
RNFL thickness, µm	20.28 (1.85)	19.76 (1.93)	−0.52 (−0.98 to −0.06)	0.03	−0.63 (−1.09 to −0.18)	0.007
INL thickness, µm	42.07 (3.89)	41.77 (4.36)	−0.29 (−1.33 to 0.74)	0.58	−0.32 (−1.35 to 0.71)	0.55

DVC_P, deep vascular plexus perfusion; SVC_P, superficial vascular plexus perfusion; DVC_D, deep vascular plexus density; SVC_D, superficial vascular plexus density.

^a^ Adjusting for age, sex, hypertension, diabetes mellitus, dyslipidemia, alcohol, smoking, and inter-eye dependencies.

**Table 3:** Retinal parameters of the AF patients according to the types of paroxysmal and sustained AF^a^.

	Paroxysmal AF vs. controls		Sustained AF vs. controls		Sustained vs. paroxysmal AF	
	β coefficient (95% CI)	*P*	β coefficient (95% CI)	*P*	β coefficient (95% CI)	*P*
Retinal microvasculature						
DVC_P	−0.05 (−0.10 to 0.001)	0.05	−0.01 (−0.05 to 0.04)	0.78	0.04 (−0.02 to 0.09)	0.171
SVC_P	−0.09 (−0.15 to −0.02)	0.01	−0.08 (−0.15 to −0.01)	0.02	0.003 (−0.07 to 0.08)	0.93
DVC_D	−0.86 (−1.95 to 0.22)	0.12	0.09 (−0.96 to 1.14)	0.87	0.84 (−0.32 to 2.01)	0.16
SVC_D	−2.04 (−3.52 to −0.56)	0.007	−1.60 (−3.12 to −0.09)	0.04	0.34 (−1.27 to 1.96)	0.68
Retinal structure						
GCIPL thickness, µm	−2.96 (−5.28 to −0.64)	0.01	−1.49 (−3.86 to −0.87)	0.22	1.44 (−1.08 to 3.96)	0.26
RNFL thickness, µm	−0.58 (−1.08 to −0.08)	0.02	−0.71 (−1.32 to −0.10)	0.02	−0.10 (−0.71 to 0.51)	0.75
INL thickness, µm	−0.63 (−1.72 to 0.47)	0.26	0.10 (−1.33 to 1.54)	0.89	0.67 (−0.74 to 2.08)	0.35

DVC_P, deep vascular plexus perfusion; SVC_P, superficial vascular plexus perfusion; DVC_D, deep vascular plexus density; SVC_D, superficial vascular plexus density.

^a^ Adjusting for age, sex, hypertension, diabetes mellitus, dyslipidemia, alcohol, smoking, and inter-eye dependencies.

### The interaction effects of LA diameter and AF subtypes in retinal parameters

In our 125 AF cohort, LA echocardiographic data were available for 120 individuals. Individuals with sustained AF had a larger LA diameter compared with paroxysmal AF [mean (SD): 43.9 (5.88) vs. 36.9 (5.52); *P* < 0.001]. The main clinical and demographic characteristics are displayed in Supplementary Table S1 and did not significantly differ between the groups.

**Figure 4:**
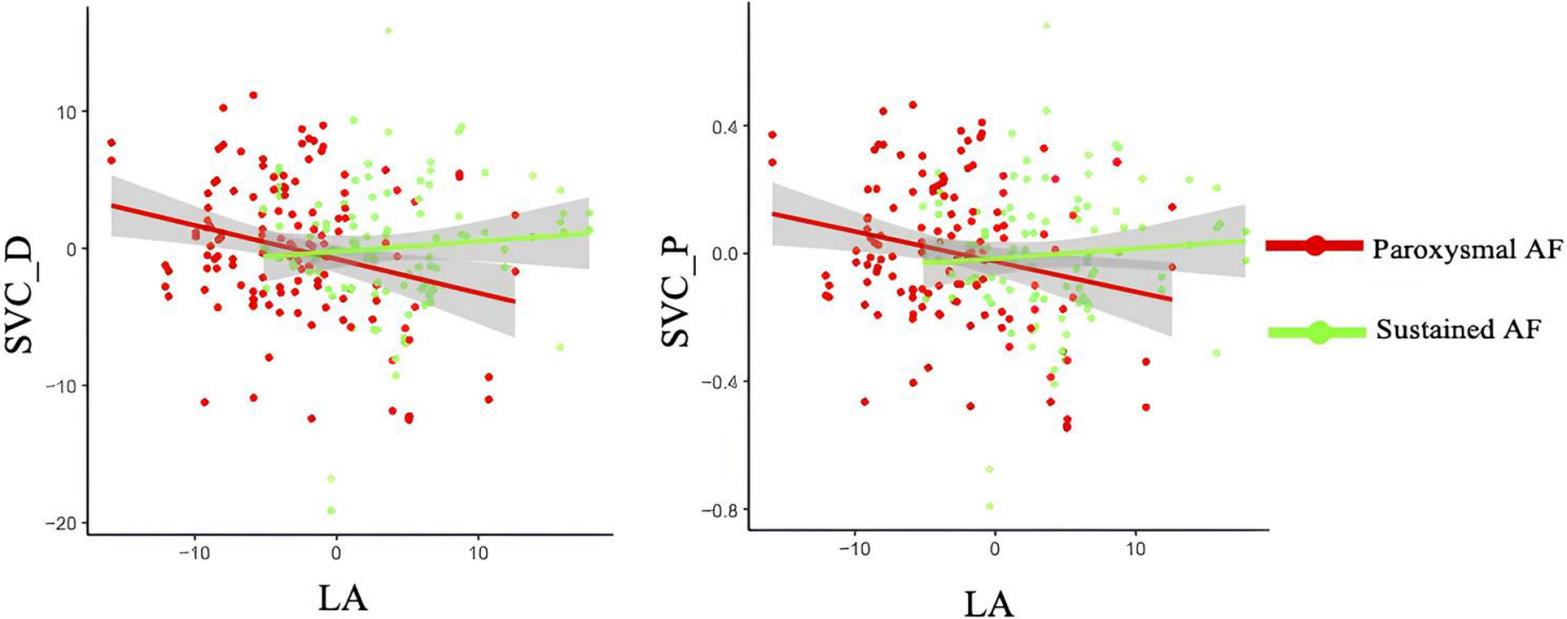
Interactions of LA diameter and AF subtypes on retinal densities and perfusion in SVC. SVC_D, superficial vascular plexus density; SVC_P, superficial vascular plexus perfusion.

When the cross-product term of AF subtypes (i.e., paroxysmal and sustained AF) and LA diameter was included in the linear regression model with each retinal parameters as the outcome, the interaction was significant for the association with the perfusion and densities in SVC in the 120 AF cohort with available LA echocardiographic data (all *P* < 0.05, Table 4 and Figure 4). No association was observed regarding the retinal vascular changes in DVC and thickness of GCIPL, RNFL, and INL (Table 4 and Supplementary Figure S1).

**Table 4:** Association of AF subtypes with retinal parameters with the interaction term AF subtypes × LA diameter^a^.

	AF subtypes × LA diameter
Outcome: DVC_P	
β (95% CI)	0.002 (0.99–1.01)
*P* for interaction	0.56
Outcome: SVC_P	
β (95% CI)	0.01 (1.00–1.02)
*P* for interaction	0.03
Outcome: DVC_D	
β (95% CI)	0.06 (0.90–1.26)
*P* for interaction	0.50
Outcome: SVC_D	
β (95% CI)	0.31 (1.07–1.7)
*P* for interaction	0.01
Outcome: GCIPL thickness, µm	
β (95% CI)	0.19 (0.84–1.74)
*P* for interaction	0.30
Outcome: RNFL thickness, µm	
β (95% CI)	0.04 (0.96–1.14)
*P* for interaction	0.33
Outcome: INL thickness, µm	
β (95% CI)	0.11 (0.92–1.35)
*P* for interaction	0.27

DVC_P, deep vascular plexus perfusion; SVC_P, superficial vascular plexus perfusion; DVC_D, deep vascular plexus density; SVC_D, superficial vascular plexus density.

^a^ Linear regression model adjusting for age, sex, hypertension, diabetes mellitus, dyslipidemia, alcohol, smoking, LA diameter, AF subtypes, and AF subtypes × LA diameter.

## Discussion

In this cross-sectional study, we observed decreased retinal vascular perfusion and densities in the SVC in individuals with AF compared with control participants. Moreover, we observed thinner GCIPL and RNFL thickness in participants with AF compared with control individuals. It indicated that objective retinal vascular and structural changes may exist in individuals with AF. On the analysis of the retinal microvasculature and structure between paroxysmal and sustained AF, no significant differences were found. However, significant interactions between LA diameter and AF subtypes with retinal microvasculature in SVC were found. Our study yielded some preliminary evidence that the differences of the retinal microvasculature in SVC between paroxysmal and sustained AF depend on the LA diameter.

Previous reports showed microvascular signs of retinopathy with AF. Using the fundus photography, Lin et al. (27) showed that retinal focal arteriole narrowing is associated with incident AF. A recent report (12) using the OCTA showed that AF individuals had a reduced density of the radial peripapillary capillaries (RPCs, capillaries around the optic nerve head) when compared with control individuals. Our current study analyzed the microvascular density, microvascular morphology, and retinal thickness in AF patients compared with control individuals; we showed that AF patients had lower SVC microvascular density, sparser SVC, and thinner RNFL and GCIPL thicknesses when compared with control individuals. The SVC contains arterioles and venules of the retina and is responsible for the arterial circulation of the retina (28). In addition, with the SVC being the entry point of blood flow into the retina, it suggested to be sensitive to ischemic changes in the retina (29). This may help explain why we found microvascular density and perfusion significantly decreased in the SVC layer rather than that in the DVC layer in the current study. Since patients with AF have reduced cardiac output ultimately resulting in reduced blood supply (ischemia) (4), we suggest that the reduced microvasculature and sparser microvasculature in the SVC of AF participants may reflect the ischemic changes associated with the disease cascade. Concerning the DVC, we did not find any significant differences between the two groups. Previous reports have shown that AF may increase the risk of retinovascular pathology via decreased blood flow. Given that the DVC is located in the deeper portion of the retina, we suggest that this microvascular plexus may not be sensitive to ischemic changes associated with AF.

Quantification of the RNFL and GCIPL thicknesses is an indicator of retinal ganglion cell integrity with the RNFL composed of retinal cell axons, while the GCIPL consists of both cell bodies and dendrites of the retinal ganglion cells (30, 31). We showed that AF participants had thinner RNFL and GCIPL thicknesses when compared with control individuals. Since the RNFL and GCIPL thicknesses reflect the neuronal integrity of the retina, we suggest that the thinner retinal structure may reflect neurodegeneration which occur in AF (32–35). On the other hand, the SVC acts as a metabolic supply of the retinal ganglion cell layer. Changes in the retinal structure (RNFL and GCIPL) seen on the OCT further complement the already established retinal vascular indicators. One may speculate that neurodegeneration may be attributed to ischemia caused by the reduced microvascular density and sparser microvasculature as seen in our current study. In addition, whether thinner retinal structures could be regarded as markers of subclinical (decreased brain volume and brain atrophy) and clinical neurodegenerative impairment (Alzheimer's disease and dementia) in AF patients is of interest to be clarified in our participants with longitudinal follow-up of brain MRI and cognitive measures.

AF commonly begins as paroxysmal, progresses over time, and then becomes sustained AF as the end result (36). During the progressive process, it leads to structural, functional, and electrical changes in the left atrium. Previous studies suggested that sustained AF has more progressive LA remodeling and dysfunction than paroxysmal AF (16, 37). This is similar to our study observing a larger LA diameter in sustained AF than in paroxysmal AF. We suggest that LA dysfunction, normally not observed in the beginning of the disease, as is seen in paroxysmal AF patients, may play a role in early identification of AF progression, which may lead to new therapies focusing on patients with “early” forms of AF.

In regard to AF subtypes, we did not find any significant differences in retinal vascular and structural parameters between paroxysmal and sustained AF. Despite this, an interaction between LA diameter and AF subtypes on retinal vascular densities and perfusion in SVC was observed in our AF cohort; it suggested that the differences of retinal vascular parameters between paroxysmal and sustained AF depended on the level of LA diameter. LA enlargement was frequently observed in patients with AF due to asynchronous atrial contraction (38); it was suggested to be linked with disrupted blood flow, endothelial damage, and microvascular dysfunction (39). In addition, LA was reported to correlate with larger retinal arteriolar branching angle (40). In the retina, decreased microvasculature and sparser microvasculature in the SVC were reported to be indicators of disturbed blood flow, tissue hypoxia, and vessel wall dysfunction. AF is also related to reduced ejection fraction and cardiac output, resulting in microvascular dysfunction (4). Given this, the interaction between LA diameter and AF subtypes with retinal vascular changes in our AF cohort suggested that AF subtypes and LA diameter jointly influenced the retinal density and morphology in SVC.

No significant interactions between LA diameter and AF subtypes on retinal microvasculature in DVC and retinal structures were found in the study. This may suggest that subclinical cardiac structure alterations (i.e., larger LA diameter) and AF were linked to suboptimal retinal arteriolar circulation instead of retinal venules and structures, supporting the hypothesis that arteriolar circulation changes may possibly be more sensitive to left atrial remodeling and AF progression. One possible explanation might be that alterations in venules and structures may have an adaptive and compensatory response, which would break down when cardiac disease becomes overt, eventually resulting in changes in venules and structures (40). Future studies are needed to validate our hypothesis and clarify the underlying mechanism.

Our study reports novel associations between AF and retinal parameters, and the differences of retinal vascular changes between paroxysmal and sustained AF depend on the LA diameter. In spite of this, we acknowledge the limitations of our study. First, the sample size of the investigated groups limited our statistical power, and we did not do multiple comparison. Second, the results were obtained from an AF cohort with Chinese, which limited the generalizability of our findings to other races, since previous studies reported racial differences in retinal vessel geometric characteristics and LA structure (20, 41). Thus, further studies with larger samples and multiple races are warranted to confirm our results. Third, other potential factors, such as systemic blood pressure (42), signal quality (43), axial length of the eyes (44), obstructive sleep apnea (45), and undiagnosed glaucoma that may have profound effects on both the structure and perfusion of the microvasculature, should be taken into account when recruiting suitable individuals. Last, the cross-sectional design of our study limited us to investigate the temporal nature of the observed associations. Further longitudinal follow-up of these participants may be helpful in providing greater insights into the association of AF with retinal microvasculature and structure.

## Conclusion

In conclusion, our data suggests that AF was linked with decreased retinal vascular densities and perfusion in SVC and thinner GCIPL and RNFL thicknesses. In addition, a significant interaction between LA diameter and AF subtypes on retinal vascular alterations in SVC was observed. It may suggest that the retinal vascular differences in AF subtypes (i.e., paroxysmal and sustained AF) depend on the LA diameter. Given our limited sample size and scarcity of reports on this topic, further longitudinal studies with larger samples may be warranted to investigate the natural history of retinal microvascular and structural changes in individuals across the clinical process of AF and AF subtypes. Such studies may clarify whether these retinal findings may be useful as biomarkers for the onset of AF or AF progression.

## Data Availability

The raw data supporting the conclusions of this article will be made available by the authors, without undue reservation.
