# Supplementary material for: Characterization of retinal microvasculature and structure in atrial fibrillation
Source: Front Cardiovasc Med. 2023 Dec 13;10:1229881. doi: 10.3389/fcvm.2023.1229881 (PMC10751341; doi:10.3389/fcvm.2023.1229881)

**Supplementary materials**

**Supplementary table I. Characteristics of AF Patients with LA Echocardiographic data according to AF Subtypes**

LA, left atrium; AF, atrial fibrillation;

**Supplementary Figure I. Interactions of LA diameter and AF subtypes on retinal vascular and structural parameters**

LA, left atrium; AF, atrial fibrillation; DVC_P, deep vascular plexus perfusion; DVC_D, deep vascular plexus density; GCIPL, ganglion cell-inner plexiform layer; RNFL, retinal nerve fiber layer;

**Supplementary table I. Characteristics of AF Patients with LA Echocardiographic data according to AF Subtypes**

|  | **All(n=120)** | **Paroxysmal AF(N=67)** | **Sustained AF(N=53)** | **P** |
| --- | --- | --- | --- | --- |
| **Age, mean (SD)** | 61.72 (8.66) | 62.45 (8.84) | 60.79 (8.41) | 0.3 |
| **Male, n(%)** | 70 (58.3) | 38 (56.7) | 32 (60.4) | 0.71 |
| **Hyperlipedemia, n(%)** | 18 (15.0) | 9 (13.4) | 9 (17.0) | 0.62 |
| **Hypertension, n(%)** | 45 (37.5) | 26 (38.8) | 19 (35.8) | 0.85 |
| **Diabetes, n (%)** | 20 (16.7) | 10 (14.9) | 10 (18.9) | 0.63 |
| **Drinking, n (%)** | 37 (30.8) | 20 (29.9) | 17 (32.1) | 0.84 |
| **Smoking, n (%)** | 35 (29.2) | 20 (29.9) | 15 (28.3) | 0.92 |
| **LA diameter(mm), mean (SD)** | 40 (6.64) | 36.9 (5.52) | 43.9 (5.88) | <0.001 |

**Supplementary Figure I. Interactions of LA diameter and AF subtypes on retinal vascular and structural parameters**


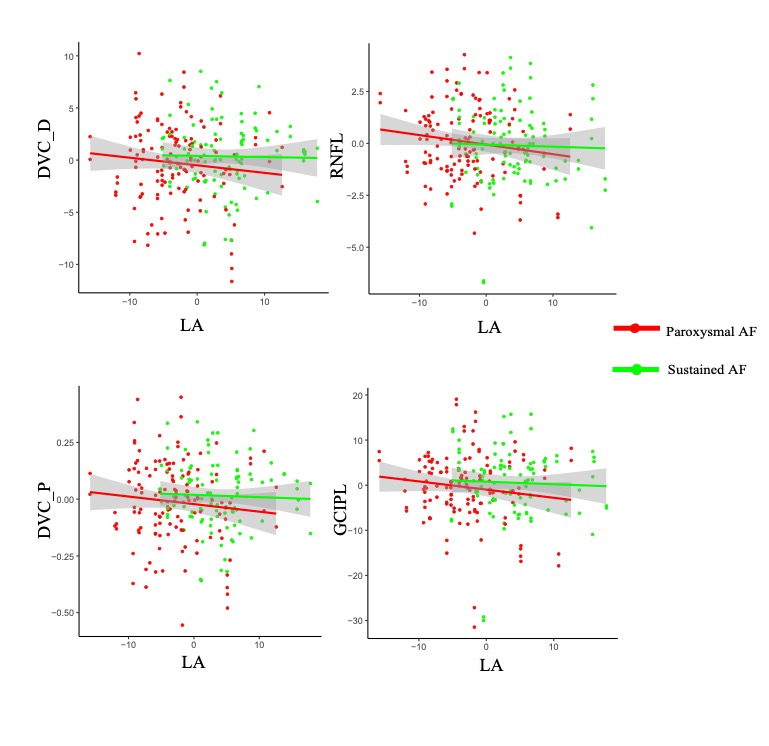

Supplement: Supplementary file 1 [file Table1.docx]
